# Privileged worlds, additivity and maximal risk

**DOI:** 10.1007/s11229-026-05756-x

**Published:** 2026-08-12

**Authors:** Martin Smith

**Affiliations:** University of Edinburgh, Edinburgh, UK

**Keywords:** Probabilistic risk, Normic risk, Unified risk, Privileged worlds, Additivity, Maximal risk

## Abstract

In ‘The unified theory of risk’ (2024) Jaakko Hirvelä and Niall Paterson consider the recent debate between the probabilistic, modal and normic theories of risk, and propose an intriguing new account – the titular *unified theory*. In this paper I focus on Hirvelä and Paterson’s criticisms of the normic theory, which are based on what they call the ‘privileged world problem’ and on the idea that risks ‘add up’. I will argue that these criticisms can, to an extent, be avoided by adopting a certain reinterpretation of the normic theory, particularly as it pertains to the notion of *maximal risk*. I then turn to the unified theory of risk and argue that, on close inspection, the theory is very similar to one of the rival theories that it purports to unify – namely, the probabilistic theory – and may share its shortcomings.

## Introduction

According to the *probabilistic* theory of risk, the risk of a proposition P is determined by the probability of P, given the background evidence – the higher the probability, the higher the risk. While this is the conventional way of thinking about risk, and has been taken for granted throughout a range of areas in which the notion of risk features, a handful of alternative theories have recently sprung up in the philosophical literature. According to the *modal* theory of risk, the risk of a proposition P is determined by the similarity of the most similar possible worlds at which the background evidence holds and P is true – the more similar these worlds, the higher the risk (see Pritchard, 2015, 2016). According to the *normic* theory of risk, the risk of a proposition P is determined by the normalcy of the most normal possible worlds at which the background evidence holds and P is true – the more normal these worlds, the higher the risk (see Ebert et al., 2020; O’Sullivan & Mace, 2024).

In ‘The unified theory of risk’ Jaakko Hirvelä and Niall Paterson argue that all of these theories are inadequate and propose a new *unified* theory of risk, which combines elements of the probabilistic and modal theories. The plan for this paper is as follows. First, I will rehearse some arguments that philosophers have given against the probabilistic theory and in favour of alternatives such as the modal and normic theories. Next, I will discuss a criticism that Hirvelä and Paterson level at the normic theory – what they call the ‘privileged world problem’ – and propose a reinterpretation of the theory that enables us to avoid the problem. I then turn to a further criticism that they raise, based on the idea that risks should ‘add up’, and argue that the proposed reinterpretation can also partially address this criticism. But the fact that normic risk is not additive is, in one sense, an integral feature of the theory. Finally, I will set out Hirvelä and Paterson’s own theory and raise a problem. On close inspection, the unified theory turns out to be very similar to the *probabilistic* theory – so much so that it may be equally vulnerable to the objections that have been raised against it. If one is persuaded by these objections, they will have reason to reject the unified theory. If one is not persuaded by these objections, they will have no reason to shift from the original probabilistic theory.

## Probability and risk

Consider a large pile of revolvers, all empty save for a single bullet in one chamber of one revolver somewhere in the pile. Suppose person A pulls a revolver from the pile at random, spins the cylinder, aims at the head of an innocent person B and is in the process of squeezing the trigger. All else equal, A’s behaviour strikes us as utterly reckless and as morally abhorrent. In this situation, it would be morally permissible for B or for a third party to use force to prevent A from pulling the trigger. If A does pull the trigger then, even if the gun doesn’t discharge, it’s clear that A has morally wronged B and, absent a strong reason or excuse, ought to be punished for their conduct (Thomson 1983; Smith 2024a).

One natural explanation for our reaction to this case is that A is subjecting B to a *high risk* of death – and this is what makes A liable to punishment or defensive action. But the probabilistic theory predicts otherwise; if the number of revolvers is very large then, according to the probabilistic theory, the risk to B is in fact very *low* and, if the pile of revolvers is large enough, could even be comparable to the risks involved in everyday activities such as driving. The modal and normic theories offer a different prediction – given the set-up, the bullet could just as easily have been in any chamber of any revolver in the pile, and it would be equally normal for the bullet to be in any chamber of any revolver in the pile. Thus there is a very similar world and a very normal world in which the bullet happens to be in the very chamber that rotates into alignment with the barrel of A’s revolver as they pull the trigger. According to the modal and normic theories, the risk to B is high (Smith 2024a, Sect.  4).

If we were witnessing these events unfold, it may be natural to reason in something like the following way: The bullet has to be in *some* chamber of *some* revolver, and it might just as well be in the chamber now sliding into alignment with the barrel, in which case B is about to be shot and killed. For the bullet to be in that chamber would require no more *explanation* that it being in any other chamber. Notice that this reasoning is unaffected by the *number* of revolvers in the pile – even though this factor is crucial when it comes to determining the probabilistic risk to B. The more revolvers there are, the more places the bullet could end up, but there is still nothing preventing it from being in the one chamber that would result in B’s death. This kind of reasoning engages directly with the normic theory of risk, on which the relevant notion of normalcy is understood in terms of a need for explanation, with abnormal events requiring more explanation than normal events (Smith, 2010, 2016, Chap. 2). In this case there would be nothing abnormal about the bullet being in the one chamber that would result in B’s death, as no special explanation would be required if it were.

Another important motivation for the modal and normic theories concerns the ‘bomb cases’ first described by Pritchard (2015). Suppose a bomb has been placed in a highly populated area and would cause significant death and devastation if it were to detonate. Consider two scenarios. In Bomb case 1 the bomb will explode if and only if one particular set of numbers comes up in a large fair lottery draw. In Bomb case 2 the bomb will explode if and only if three highly unlikely events obtain; the weakest horse in the field at the Grand National must win the race by at least ten furlongs, the worst team remaining in the FA Cup draw must beat the best team remaining by at least ten goals, and the King must spontaneously choose to speak a complete sentence of Polish during his next public speech.

According to Pritchard, the risk of the bomb exploding is higher in Bomb 1 than Bomb 2 and, if given a choice, we ought to prefer Bomb 2 over Bomb 1. To generate a problem for the probabilistic theory, we need to stipulate that the lottery in Bomb 1 has enough possible outcomes to ensure that the probability of the bomb going off is equal to the probability in Bomb 2. On the probabilistic theory, the risks in the two cases would then be equivalent and, all else equal, we ought to be indifferent between them. The size of the lottery required will depend on just how improbable we take the three triggering events in Bomb 2 to be. Pritchard suggests a figure of one in 14 million – but if we think this figure is too high (as it arguably is – see Ebert et al., 2020, pp. 436-437), then we need only imagine a larger lottery. Pritchard supposes that if the lottery in Bomb 1 is fair and is assured to have some outcome, then the number of possible outcomes, and the associated improbability of the bomb going off, won’t affect our judgment that this case involves a higher risk than Bomb 2 (Pritchard, 2015, pp. 443-444).

The modal and normic theories of risk make a different prediction about the two bomb cases. For any given lottery outcome, there will be very similar worlds and very normal worlds in which that outcome obtains. No one lottery outcome would require more explanation than any other – that’s part of what it is for the lottery to be fair. In contrast, the most similar/normal worlds in which the weakest horse wins by at least ten furlongs, the worst team beats the best team by at least ten goals, and the King speaks Polish in his next public speech will be very dissimilar/abnormal. Any one of these events would require significant explanation if it were to occur. On the modal and normic theories, there is a high risk of the bomb going off in Bomb 1 while the risk of the bomb going off in Bomb 2 is significantly lower. And this will hold true irrespective of the size of the lottery involved in Bomb 1 – unlike the probabilistic risks, the modal and normic risks don’t depend on how many possible lottery outcomes there are.

In this section I’ve outlined two arguments against the probabilistic theory of risk and in favour of alternatives such as the modal or normic theories. Hirvelä and Paterson don’t discuss the revolver example (though see Hirvelä, 2025, p. 339), but they do endorse Pritchard’s judgment about the bomb cases (Hirvelä & Paterson, 2024, Sect.  2). More generally, Hirvelä and Paterson seem sympathetic to the standard arguments against the probabilistic theory – otherwise there would be no reason to seek a *new* theory. As suggested above, these arguments will also prove to be important for assessing the new theory that Hirvelä and Paterson propose.

Before moving on, I will briefly note two different ways of framing the debate between alternative accounts of risk. While Ebert et al. (2020) and O’Sullivan and Mace (2024) give arguments in support of the normic theory, they favour a *pluralist* view on which the probabilistic theory also represents a perfectly legitimate conception of risk (see also Ebert and Pedersen, forthcoming). Pritchard, particularly in his earlier work on risk, appears to operate within a *monist* framework, on which there is room for only one correct account of risk, which he takes to be the modal theory^1^. Hirvelä and Paterson are also sympathetic to monism and present the unified theory as an ‘all-purpose’ account of risk, which doesn’t need to be supplemented by any further theories (Hirvelä & Paterson, 2024, Sect.  4). The monism vs. pluralism issue won’t assume much significance here, but is something I will return to from time to time.

## The privileged world problem

One objection to the modal theory of risk begins with the observation that no other world can be more similar to the actual world than it is to itself. As a result, any proposition which is true at the actual world must have a maximal modal risk. Amongst other things, this means that, on the modal theory, one cannot assess the risk of a proposition without taking a view on its truth^2^. The normic theory avoids this result, since other worlds may be rated as more normal than the actual world. According to Hirvelä and Paterson, however, this objection to the modal theory is really an instance of a more general problem which *does* apply to the normic theory as well – the existence of ‘privileged’ possible worlds that dictate maximal risk. Recall that the normic risk of P is greater than the normic risk of Q just in case the most normal worlds at which the background evidence holds and P is true are more normal than the most normal worlds at which the background evidence holds and Q is true. As a result, if P is true at one of the *most* normal worlds at which the evidence holds then there is no scope for any other proposition to have a higher normic risk. Any proposition which is true at a maximally normal world must have a maximal normic risk. This is what Hirvelä and Paterson refer to as the *privileged world problem*^3^.

Let W be the set of possible worlds in which the background evidence holds. To use a familiar metaphor, we can imagine these worlds as points in space, with proximity to the centre representing how normal they are. We can then imagine the worlds organised into a series of concentric spheres – the smallest, most central, sphere will contain those worlds that are maximally normal, the next sphere will incorporate the worlds that are almost but not quite so normal and so on. Normalcy spheres are required to be nested – for any two spheres, one must be a subset of the other – and to exhaust the set W – every possible world in W will be a member of some normalcy sphere^4^. If we are dealing with a finite set of worlds, the spheres can be ordered and numbered, providing a means of *measuring* normic risk^5^.

Let the degree of abnormality of a proposition P be equal to the total number of normalcy spheres throughout which P is false – the number of spheres that we have to pass through before we find worlds at which P holds (Smith, 2016, Sect.  8.3, 2024a, Sect.  4). If P has a degree of 2 then there are exactly two normalcy spheres throughout which P is false, if P has a degree of 7 then there are exactly seven normalcy spheres throughout which P is false etc. If P is false throughout every normalcy sphere, then it might be assigned a degree of ∞^6^. Remember that these are degrees of *abnormality* – the lower the number, the more normal P is and the *higher* the normic risk that P is true. This gives us another angle on the privileged world problem. The abnormality of P will be at a minimum, and the normic risk at a maximum, when there are *no* normalcy spheres throughout which P is false – i.e. when P is true at some worlds in the smallest sphere (Hirvelä & Paterson, 2024, pp. 9-10)^7^.

The very idea of privileged worlds that determine maximal risk might already seem somewhat counterintuitive – it’s certainly very alien to the probabilistic theory, on which it’s more natural to think of the risk of a proposition as corresponding to the *proportion* of worlds at which it holds. But Hirvelä and Paterson go on to derive some further counterintuitive consequences from the existence of privileged worlds (Hirvelä & Paterson, 2024, Sect.  3). Consider the following principle: A proposition that is entailed by the evidence should have a higher risk than a proposition that is not entailed by the evidence. If W is the set of worlds at which the evidence holds, then a proposition that is entailed by the evidence will be true at every world in W. This gives us another way of stating the principle: A proposition that is true at every possible world should have a higher risk than a proposition that is false at some possible worlds (Hirvelä & Paterson, 2024, p. 8). But the existence of privileged worlds means that the normic theory will violate this principle. While a proposition that is true at every possible world will be of maximal normic risk, this is also true of a proposition that holds at some of the maximally normal worlds, even if it’s false at many other worlds. In the extreme case this would even apply to a proposition that is true at *one* maximally normal world and false at *every other world* in W.

If a proposition is true at just one world then it represents a complete specification of the extra-evidential facts – the facts which go beyond the background evidence. And if this world is a maximally normal one, then that specification involves no surprises or abnormalities – everything unfolds in a mundane way. While this proposition may be exceedingly unlikely in probabilistic terms^8^, this is just a function of the sheer *number* of contingent facts that it specifies, and doesn’t reflect anything about the nature of these facts, which are individually banal. On a normic way of thinking, there *is* a certain risk to a proposition like this. After all, there must be *some* correct way of filling in the extra-evidential facts and it could just as well be *this* way as any other (compare: the bullet has to be in some chamber of some revolver, and it might just as well be in the chamber now sliding into alignment with the barrel). But even when thinking in this way, the idea that this proposition should have the *same risk* as something that holds at *every* possible world seems difficult to accept.

Another consequence of the existence of privileged worlds is that a proposition and its negation could *both* count as being maximally risky (Hirvelä & Paterson, 2024, p. 9). There must be propositions which separate the maximally normal worlds – that is, propositions which are true at some maximally normal worlds and not at others. Let P be one such proposition. Since the maximally normal worlds are privileged, it immediately follows that both P and ∼P have maximal normic risk^9^. This might seem particularly strange from the perspective of the probabilistic theory, on which the risk of P and the risk of ∼P are complementary – raising one means lowering the other. We shouldn’t expect normic risk to work in exactly the same way as probabilistic risk of course, but perhaps this is just too great a departure.

## Two-sided normic risk

Before proceeding, we should note that there is something awkward about the notion of ‘maximal risk’. While we would often describe things as being ‘high risk’ or ‘low risk’ or even ‘no risk’, to say that some outcome has ‘maximal risk’ would not fit so well into ordinary conversation and sounds more like a technical claim. Without any obvious interpretive steer, though, I think that most people would take this to mean that the outcome in question is *ensured* or *guaranteed*. Swallowing a lethal dose of cyanide or playing Russian roulette with a bullet in every chamber might be thought to involve a maximal risk of death in this sense. But on the normic theory, as it’s currently set up, the notion of maximal risk is not limited to cases like these, and will also apply to cases in which a negative outcome is far from guaranteed. I think this is, in a way, the basic source of the privileged world problem – a mismatch between the point at which the normic risk scale ends and what ‘maximal risk’ would most naturally be taken to mean. But the decision to end the normic risk scale at this point – when a proposition is true at some worlds in the smallest normalcy sphere – is not forced upon us. The scale can continue.

If a proposition is true at *all* of the worlds in the smallest normalcy sphere then that can be thought of as representing a *higher* normic risk than a proposition that is only true at some of these worlds. And if a proposition is true at all of the worlds in the second normalcy sphere then this will represent a higher risk again and so on. Put differently: If the *falsity* of a proposition would require explanation then this can be thought of as representing a higher normic risk than a proposition whose truth and falsity are on an explanatory par. And if the falsity of a proposition would require even more explanation then this will represent a higher risk again and so on. There is another side to the normic risk scale.

Compare the following two scenarios. In the first case, you have just woken up, the curtains are drawn and you have no information about the weather. In the second case, you walk over to the curtains and can hear what sound like water droplets striking the glass behind. You then walk to the front door and see a soaked jacket, boots and umbrella left by someone who has just come in. On the original way of thinking, the normic risk that it is currently raining would be maximal in both of these cases. In neither case would the possibility of rain be abnormal, or require a special explanation, in light of your evidence. But on the new ‘two-sided’ way of thinking, the normic risk of rain is *higher* in the second case than the first – in the second case we would need a special explanation in the event that it is *not* raining.

When a proposition is true at some of the most normal worlds and not at others, this might be regarded as the *midpoint* on the normic risk scale – the normic equivalent of a probability of 0.5. This is the point at which the truth and falsity of the proposition should be regarded as equally *normal*, in the same way that the truth and falsity of a proposition with probability 0.5 should be regarded as equally *likely*. In Bomb 1 the normic risk of the bomb detonating should no longer be regarded as maximal. Rather, this will represent a middle normic risk, as there are maximally normal worlds at which the bomb detonates and maximally normal worlds at which it doesn’t. But this is still significantly higher than the normic risk of a detonation in Bomb 2, which only occurs at highly abnormal worlds. Similar remarks apply to the revolver case – there will be some maximally normal worlds at which B is killed and some at which B survives. But when it comes to an extremely adverse outcome, such as a death or a bomb detonating in a public area, even a middle normic risk should be considered very *high*, as would a 0.5 probability. So the claim that Bomb 1 and the revolver case involve a high normic risk still stands – it’s just that these scenarios will no longer be classified as involving *maximal* normic risk.

Let the *degree of entrenchment* of a proposition P be equal to the total number of normalcy spheres throughout which P is true – the number of spheres that we have to pass through before we find worlds at which P fails to hold (Smith, 2016, Sect.  8.3). If P is true throughout every normalcy sphere, then it might be assigned a degree of ∞. What I propose is that the normic risk of P be measured by the degree of entrenchment of P minus the degree of abnormality of P – in effect, the total number of normalcy spheres throughout which P is true minus the total number of normalcy spheres throughout which P is false. This gives us a scale running from -∞ to ∞ with -∞ representing minimal normic risk and ∞ representing maximal normic risk.

No proposition can have a positive degree of entrenchment *and* a positive degree of abnormality – at least one of these values must be 0. Since normalcy spheres are nested, it is impossible for a proposition to be true throughout one normalcy sphere and false throughout another. If there is a normalcy sphere throughout which P is false, then P will have a negative normic risk, with the degree indicating the number of normalcy spheres throughout which its falsity extends. If there is a normalcy sphere throughout which P is true, then P will have a positive normic risk, with the degree indicating the number of normalcy spheres throughout which its truth extends. If P is not true or false throughout any normalcy sphere – that is, true at some worlds in the smallest sphere and false in others – then it will have the middle normic risk of 0^10^.

This might seem like a significant shift from the standard one-sided picture of normic risk. But in one way the change is conventional rather than substantial. The comparative normalcy ordering of possible worlds is exactly as before – it’s just that we are now using more of the information that it encodes in order to generate another side to the normic risk scale. But this does lead to a change in some of the fundamental aspects of the theory – such as the clause for comparing risks. The normic risk of a proposition P was said to be higher than the normic risk of a proposition Q just in case the most normal worlds at which P is true are more normal than the most normal worlds at which Q is true. This rule for normic risk comparisons will still hold for negative normic risks – risks that are below the midpoint – but for positive normic risks we will need to use a new rule:

The normic risk of P is higher than the normic risk of Q just in case the most normal worlds at which P is false are less normal than the most normal worlds at which Q is false.

And we can give a fully general rule, which covers the whole scale, by using a disjunctive condition:

The normic risk of P is higher than the normic risk of Q just in case *either* the most normal worlds at which P is true are more normal than the most normal worlds at which Q is true or the most normal worlds at which P is false are less normal than the most normal worlds at which Q is false.

Once we move to a two-sided interpretation of normic risk, there are no longer any privileged worlds – no longer any worlds at which truth suffices for maximal risk. The maximally normal worlds will now have a ‘semi-privileged’ status – if P is true at a maximally normal world it follows that the normic risk of P is no lower than the midpoint. But this does not lead to the same problematic consequences. If a proposition is entailed by the evidence and, thus, true at all worlds in W it will now have a higher normic risk than a proposition which is not entailed by the evidence and, thus, false at some worlds in W. If a proposition is true at all worlds in W then it will be true throughout every normalcy sphere and will have a normic risk of ∞. If a proposition is false at some world in W then it cannot be true throughout every normalcy sphere and will not have a normic risk of ∞. If a proposition is true at just one maximally normal world, as imagined in Sect.  3, it will have a normic risk of 0.

On the new interpretation, maximal and minimal normic risk will effectively coincide with maximal and minimal probabilistic risk. The only propositions which have maximal normic risk are those that are true at every world in W and the only propositions which have minimal normic risk are those that are not true at any world in W. If W is finite, and every world has some positive probability of being actual, then the only propositions which have maximal probabilistic risk are those that are true at every world in W and the only propositions which have minimal probabilistic risk are those that are not true at any world in W. The normic risk scale and the probabilistic risk scale will have the same endpoints, but represent different paths between them.

On the new interpretation, a proposition and its negation cannot both have maximal normic risk. If P is true at some maximally normal worlds and false at others this will now represent a situation in which P and ∼P both have the middle normic risk of 0. Normic risk will now satisfy a kind of complementarity, in that the normic risk of P and the normic risk of ∼P must sum to 0. The normic risk of P is equal to the total number of spheres throughout which P is true minus the total number of spheres throughout which P is false, and the normic risk of ∼P is simply the reverse; the total number of spheres throughout which P is false minus the total number of spheres throughout which P is true. There is, then, a sense in which raising the risk of P involves lowering the risk of ∼P and vice versa^11^. In a way, the two sides of the normic risk scale are just mirror images of one another, with each proposition and its negation being equidistant from the midpoint. As noted above, constructing the two-sided scale doesn’t require any additional information or content – it’s just a new way of representing the information already contained in the negative (or positive) side.

## Do risks ‘add up’?

Another objection that Hirvelä and Paterson level at the normic theory is based on the idea that risks should *add up*. Suppose we are about to roll a six-sided die and two people have placed bets on the outcome. The first person will lose the bet if the die lands on 6 while the second person will lose if the die lands on 5 *or* 6. Surely the second person has a *higher* risk of losing their bet – the risk that the die will land on 5 or 6 is equal to the risk that it will land on 5 *plus* the risk that it will land on 6 (Hirvelä & Paterson, 2024, p. 10). Probabilities satisfy the *additivity* axiom which states that, if P and Q are exclusive propositions – that is, propositions which cannot both be true – then the probability of P ∨ Q is equal to the probability of P plus the probability of Q. If we suppose that logically equivalent propositions must have the same probability, it follows that, for any P and Q, the probability of P ∨ Q is equal to the probability of P plus the probability of Q minus the probability of P ∧ Q^12^. As a result, the probability of P ∨ Q will almost always be larger than the probability of P and the probability of Q (the only exception being when one of these is equal to the probability of P ∧ Q).

Normic risk behaves differently. Indeed normic risk validates the following inference pattern, sometimes called ‘checklist reasoning’ (Ebert et al., 2020, Sect.  5):

1. The risk of P is low
2. The risk of Q is low
3. Therefore the risk of P ∨ Q is low

If the risk of P is low and the risk of Q is low then, on the normic theory, this means that the most normal worlds at which P holds are highly abnormal and the most normal worlds at which Q holds are highly abnormal. But any world at which P ∨ Q holds must either be a world at which P holds or a world at which Q holds. As a result, the most normal worlds at which P ∨ Q holds must also be highly abnormal, meaning that the risk of P ∨ Q is also low.

Think about the process of going through a safety checklist – if you’re about to embark on a long car journey you might check the petrol and the oil and the tyre pressure etc. No matter how stringent your checks, you won’t be able to completely rule out an adverse outcome – like the car running out of petrol or a tyre going flat. But provided the petrol gauge indicates a full tank, and the pressure readings are all normal etc. then the risk of these outcomes will be low enough for you to tick them off the list. If you have an exhaustive list and you’ve ticked everything off and you conclude, on this basis, that there’s a low risk of anything going wrong on the trip then you are using checklist reasoning.

As well as being something that we might use in everyday risk assessments, checklist reasoning appears to be presupposed by the practice of de minimis risk management. Suppose we are considering the adverse outcomes that could potentially result from a new policy or decision. Roughly speaking, de minimis risk management involves the use of a low risk threshold to sort these possibilities into two categories. Those that fall below the threshold are ignored while those that are above the threshold are subject to preventive and/or mitigating measures. The basic motivation is clear – time and resources are limited, and it is better that they be expended on addressing larger risks than squandered on small ones.

If checklist reasoning is invalid, however, then we could find ourselves in a situation in which the risk of P and the risk of Q fall below the de minimis threshold while the risk of P ∨ Q is above it. If P and Q are below the de minimis threshold then de minimis risk management tells us that we are allowed to ignore P and ignore Q. But if we have ignored both P and Q then we have ignored P ∨ Q, which is something we are *not* allowed to do. Alternately, if P ∨ Q is above the threshold then de minimis risk management tells us that we must take measures against it. But we can’t take measures against P ∨ Q without taking measures against P or taking measures against Q – which are things we are *not* required to do. Without checklist reasoning it looks as though de minimis risk management gives inconsistent recommendations.

For these and other reasons, the validation of checklist reasoning is sometimes presented as an advantage of the normic theory (Ebert et al., 2020). As Hirvelä and Paterson emphasise though, the normic theory will in fact validate something much *stronger* than mere checklist reasoning:

1. The risk of P is n
2. The risk of Q is n
3. Therefore the risk of P ∨ Q is n

This is the same basic pattern as checklist reasoning, except that it’s not limited to *low* risks, but applies to any level of risk whatsoever. Hirvelä and Paterson call this *generalised checklist reasoning* (Hirvelä & Paterson, 2024, p. 10).

Generalised checklist reasoning is not needed to make sense of de minimis risk management or everyday risk assessments, and it seems, in general, to be a lot more suspect. Consider a case involving *high* risks. Suppose the petrol gauge indicates that the tank is almost empty and the pressure readings suggest that one of the tyres has a slow leak. If you were to set out on a long driving trip nonetheless, then there is a high risk of running out of petrol and a high risk of a flat tyre, but surely the risk that *something* will go wrong would be even higher still – contrary to generalised checklist reasoning. It’s reckless to ignore a petrol gauge showing near empty and it’s reckless to ignore a pressure reading that indicates a slow leak, but surely it’s even *more* reckless ignore both of these things.

Once we shift to a two-sided interpretation of normic risk, however, generalised checklist reasoning is no longer valid. In particular, the reasoning will not go through for any *n* ≥ 0. Even if P and Q are each false at certain worlds in a given normalcy sphere, P ∨ Q could still be true throughout that sphere. That is, Q could be true at the worlds at which P is false and P could be true at the worlds at which Q is false. Therefore, if n is the total number of spheres throughout which P is true and the total number of spheres throughout which Q is true it doesn’t follow that n is the total number of spheres throughout which P ∨ Q is true. P ∨ Q can have a higher degree of entrenchment than both P and Q.

Suppose again that the petrol gauge shows close to empty and the pressure gauge indicates a slow leak in one of the tyres. If you embark on your long journey there is a high normic risk that you will run out of petrol and a high normic risk that you will get a flat tyre. If you don’t run out of petrol, there would have to be some explanation for this, such as a problem with the petrol gauge. If you don’t get a flat tyre, there would have to be some explanation for this, such as a problem with the pressure gauge. But the normic risk that *one of these things will happen* would be higher still. If neither of these things happened then there would need to be *two* explanations. While we have to travel through n spheres to reach worlds at which you don’t run out of petrol and n spheres to reach worlds at which you don’t get a flat tyre, we would have to travel through more spheres to reach worlds at which neither of these things happen.

But generalised checklist reasoning will still go through for any *n* < 0. If P and Q are both false throughout a given normalcy sphere then P ∨ Q must also be false throughout that sphere. If P ∨ Q were true at some world in that sphere then either P or Q would have to be true at that world. Therefore, if n is the total number of spheres throughout which P is false and the total number of spheres throughout which Q is false, it must also be the total number of spheres throughout which P ∨ Q is false. P ∨ Q cannot have a lower degree of abnormality than both P and Q. As a result, the original checklist reasoning pattern remains valid, provided ‘low risk’ is understood in terms of a threshold that is set below the midpoint^13^.

While a two-sided interpretation of normic risk will invalidate generalised checklist reasoning, it doesn’t give us the additivity property, or anything approaching it. If P and Q are exclusive propositions, it can still be the case that the normic risk of P ∨ Q is no higher than the normic risk of P and the normic risk of Q. This is clearly illustrated by the dice example from the start of the section. Rolling a 5, rolling a 6 and rolling a 5-or-6 would all have a middle normic risk. Hirvelä and Paterson take it to be obvious that risks add up – that the risk of a disjunction is generally higher than the risks of the disjuncts – and treat this as a kind of datum that any theory of risk must capture. I see things differently; I think it’s obvious that *probabilities* add up, and it’s obvious that probability represents one legitimate way of thinking about risk. But in assuming that *any* legitimate account of risk must respect the additivity property, Hirvelä and Paterson come close to begging the question against the normic theory, particularly if it is embedded within a risk pluralism.

On the normic theory, a risk value that is at or below the midpoint reflects the *abnormality* of a proposition P. The lower the value the more abnormal the truth of P would be. This, in turn, reflects how much *explanation* the truth of P would require. The lower the value the more explanation would be needed if P were true. Could the truth of a disjunction P ∨ Q require *less* explanation than the truth of P and the truth of Q? It seems not – after all, the only way in which P ∨ Q can be true is if either P is true or Q is true. If P would require a great deal of explanation and Q would require a great deal of explanation then so too would P ∨ Q (Smith 2024b, Sect.  4). There’s no temptation to think that normalcy, when understood in this explanatory way, should satisfy the additivity property. One might claim that normalcy, so understood, doesn’t connect with any viable conception of risk – but that, of course, is the very question at issue.

Furthermore, the idea that risks add up is arguably in tension with the primary *motivations* for the normic theory – that is, with the claim that the revolver case and Bomb case 1 involve high risk. Both of these cases involve a large number of possibilities which appear to be of equal risk; in the revolver case there are a large number of chambers in which the bullet could be (one of which would result in B’s death) and in Bomb 1 there are a large number of possible lottery outcomes (one of which would result in the bomb detonating). If risks add up then they also *divide*, which means that the more equal-risk possibilities there are, the lower the risk of each one. I will return to this in the next section.

## The unified theory of risk

Hirvelä and Paterson’s unified theory of risk^14^ is based on the idea that the risk of a proposition should be determined both by the proportion of possible worlds at which it’s true and by the similarity of the possible worlds at which it’s true. These elements are combined via the following formal model. We begin with a set of possible worlds W which could, once again, be thought of as the set of worlds consistent with a background body of evidence. Hirvelä and Paterson assume that W is finite. Next we introduce a function S which assigns a *similarity score* to each pair of worlds in W. Similarity scores are positive real numbers in the unit interval with 1 representing maximal similarity.

We can now calculate a *unified risk score* for a proposition P relative to a world w:

$$\:{R}_{w}\left(P\right)=\sum\limits_{x{\in}W,\:x\models\:P}S(w,x)/\sum\limits_{x\in{W}}S(w,x)$$

Less formally, to find the unified risk score for a proposition P at a world w we first sum the similarity scores of the worlds at which P is true and then divide this by the sum of the similarity scores of all worlds in W^15^. This will give us a real number in the unit interval, which partly reflects the proportion of worlds at which P is true, and partly reflects the average similarity of the worlds at which P is true as compared with the average similarity of all worlds. The higher the risk score, the greater the risk, with the risk being maximal when the score is 1 and minimal when the score is 0.

Risks, on the unified theory, will add up. In fact, given the above formula, unified risk scores satisfy the additivity property from the last section: For all worlds w and propositions P and Q, if P and Q are exclusive then R_w_(P ∨ Q) = R_w_(P) + R_w_(Q). It’s important to note that unified risk scores will also satisfy the other two axioms for a probability function; *nonnegativity* and *normalisation*. That is, risk scores must always be greater than or equal to 0 and the risk score of a proposition which holds at all worlds in W must be equal to 1 (Hirvelä & Paterson, 2024, Sect.  4). In one sense unified risk scores *are* probabilities – R_w_ meets all the conditions for a probability function (see also Ebert and Pedersen, forthcoming, p. 21).

This is not to say, however, that the unified theory of risk and the probabilistic theory of risk amount to the same thing. There are many probability functions that we can define over the set of propositions, and it may be that probabilistic risks are associated with a distinct function. One natural thought is that, on the probabilistic theory, world similarity should play no role in determining risk – so we might ignore the similarity scores and assign every world in W the same weight. In this case the probabilistic risk of P (at any world) will be equal to the proportion of worlds at which P holds – we might write this as ‘Pr(P)’. The probabilistic risk of a proposition, defined in this way, may be quite different from its unified risk – that is, Pr(P) may be quite different from R_w_(P). If, for instance, a proposition holds at a large number of worlds, but they all happen to be relatively dissimilar to w, then it may have a high probabilistic risk and a low unified risk at w.

So unified risks and probabilistic risks need not be the same – but they *are* subject to the same formal constraints. As a result, if a case involves broadly formal considerations that force probabilistic risks to take on particular values, then they will force unified risks to take the very same values. Suppose we have a set of propositions P_1_, P_2_, …, P_n_ that are exclusive and exhaustive – that is, exactly one of these propositions is true at each world in W, and the others are false. Since P_1_, P_2_, …, P_n_ are exclusive it follows, by additivity, that R_w_(P_1_ ∨ P_2_ ∨ … ∨ P_n_) = Σ_1≤i≤n_ R_w_(P_i_). Since P_1_, P_2_, …, P_n_ are exhaustive it follows, by normalisation, that R_w_(P_1_ ∨ P_2_ ∨ … ∨ P_n_) = 1. We have it that Σ_1≤i≤n_ R_w_(P_i_) = Σ_1≤i≤n_ Pr(P_i_) = 1. If these propositions all have an *equal* risk it then follows that R_w_(P_1_) = R_w_(P_2_) = … = R_w_(P_n_) and Pr(P_1_) = Pr(P_2_) = … = Pr(P_n_) in which case, for all i, 1≤i≤n R_w_(P_i_) = Pr(P_i_) = 1/n. Put less formally, because the unified risks of the P_i_s are equal, and the probabilistic risks of the P_i_s are equal, and the unified risks of the P_i_s and the probabilistic risks of the P_i_s must sum to the same value – i.e. 1 – it follows that the unified risk of any P_i_ must be equal to the probabilistic risk of that P_i_.

While unified risks and probabilistic risks can often come apart, there are some cases, with a particular formal structure, in which they are forced to coincide. What is more significant is that the very cases that have been used to criticise the probabilistic theory of risk – such as the revolver case and Bomb 1 – would appear to be of precisely this structure. In the revolver case, we know that there is a bullet in some chamber of some revolver. Thus we have an exclusive and exhaustive set of propositions describing the possible locations of the bullet, including the chamber that is about to slide into alignment with the barrel of the revolver that A is holding. Given the set-up, it’s natural to think that these propositions are all of equal risk. After all, the evidence doesn’t favour any one of these propositions over any other. In this case, the unified risk of B being killed is the same as the probabilistic risk of B being killed. If the latter is very low then so is the former.

Hirvelä and Paterson might reply that the risks of these propositions are not equal – there is a higher risk of the bullet being in the chamber that it’s actually in, because the actual world should have a higher similarity score than all others^16^. But this will not deliver a better result. The unified risk scores of these propositions must still sum to 1 – so if the true member of the set has a higher score, then the others must each have a slightly lower score to compensate for this. If the gun doesn’t discharge – so the bullet is in some other chamber – then, on the present proposal, the unified risk of B being killed would have been even *lower* than the probabilistic risk. And yet, as noted in Sect.  2, there is a strong impression that A’s actions are morally abhorrent and that A is deserving of punishment. The natural explanation is that this is due to the *risk* that A imposed on B. But the unified theory can no more accommodate this judgment than the probabilistic theory.

Similar considerations apply in Bomb 1. Consider the propositions describing all of the possible lottery outcomes – ‘7, 32, 14, 8, 11, 4’ are the winning numbers, ‘27, 2, 3, 17, 21, 30’ are the winning numbers, and so on … one of which will be associated with the bomb going off. We know that the lottery must have some outcome, and will only have one outcome, and we know that the risk of any one outcome is equal to the risk of any other – this is part of what it is for the lottery to be fair. As a result, these propositions are exclusive and exhaustive and of equal risk, in which case the unified risks and the probabilistic risks converge. In particular, the unified risk of the bomb exploding will be equal to the probabilistic risk of the bomb exploding^17^. Bomb 2, on the other hand, would seem to be a case in which the unified risks and probabilistic risks diverge. If the three events that would trigger the detonation in Bomb 2 only occur at relatively dissimilar worlds then the unified risk of the bomb exploding may be significantly lower than its probabilistic risk. This will give us the desired result when the probabilities are equal – the risk of a detonation will be higher in Bomb 1 than Bomb 2. But, as noted above, Pritchard’s claim about the bomb cases is much more general than this – that any lottery-style bomb case should be judged to be higher risk than Bomb 2. This is something that the unified theory cannot deliver. The unified risk of a detonation in Bomb 2 must have *some* positive value and, by increasing the size of the lottery, there will be a point at which the risk in Bomb 1 drops below it. The dissimilarity of the worlds in which the bomb detonates in Bomb 2 can be offset by shrinking the proportion of worlds in which the bomb detonates in Bomb 1 (for related discussion see Ebert and Pedersen, forthcoming, p. 21)^18^.

So the arguments against the probabilistic theory, set out in Sect.  2, also apply to the unified theory. The worry for the unified theory is not that these arguments are decisive (in so far as I accept that probability gives us one legitimate conception of risk, I think there is a sense in which the arguments are *not* decisive). Rather, the worry is precisely that the arguments apply *equally* to the two theories. The probabilistic theory is the received view, and the starting point for any theorising about risk. If we are working within a monist framework then the onus is on anyone who would reject the probabilistic theory in favour of an alternative – and the defender of the unified theory will be hard pressed to discharge this onus. If we are working within a pluralist framework, then the probabilistic theory should have a presumptive place in the plurality, and it would be redundant to give the unified theory a further place alongside it.

## Conclusion

In this paper I have proposed a reinterpretation of the normic theory of risk, adding a second side to the normic risk scale. Some situations that would have been previously characterised as involving a maximal normic risk will now be located at the midpoint of the new two-sided scale. Amongst other things, this removes the existence of ‘privileged worlds’ in Hirvelä and Paterson’s sense and invalidates the ‘generalised checklist reasoning’ pattern that they identify. While this reinterpretation does not secure an additivity property for normic risk, I have argued that the failure of additivity is inextricably linked with the original motivations for the theory and, indeed, with the original motivations for moving beyond the probabilistic conception of risk. This is illustrated by Hirvelä and Paterson’s unified theory of risk, which satisfies additivity but, I have argued, is unable to do justice to these motivations.

## Data Availability

Not applicable.

## Footnotes

1 Pritchard, at any rate, does not seem to allow any place for the probabilistic theory of risk, describing it as ‘fundamentally misguided’ (Pritchard, 2015, p. 436). But he does grant that probabilities can still be useful for assessing risk, since they may be correlated with facts about world similarity (Pritchard, 2015, p. 449).

2 More precisely, one cannot judge a proposition to be low or medium risk without assuming that the proposition is false. There are some propositions – like the proposition that the bomb detonates in Bomb 1 – that can be judged to be high risk irrespective of whether they are true or false. Versions of this objection have been given by Ebert et al. (2020, Sect.  4), Gardiner (2020, Sect.  5), Fratantonio (2021, Sect.  4.1), Newton (2022, Sect.  3) and Smith (2023, Sect.  1).

3 Both the normic theory and the modal theory predict the existence of privileged worlds, with the modal theory having the additional feature that the actual world must count as privileged.

4 If we have an infinite set of worlds, and of normalcy spheres, then we might impose the additional constraint of closure under (nonempty) unions and intersections; the union and the intersection of any (nonempty) set of normalcy spheres must themselves be normalcy spheres. For finite sets of normalcy spheres this is already guaranteed by the existing constraints.

5 This also holds for an infinite W, provided we impose the world-normalcy equivalent of Lewis’s *limit assumption* (see Lewis, 1973, Sect.  1.4, Smith, 2016, Chap. 8) – in short, the assumption that every set of worlds has maximally normal members. If we have an infinite set of worlds, and an infinite set of normalcy spheres then, without this assumption, there is no guarantee that each sphere will have a unique successor. In saying that this gives us a way of ‘measuring’ normic risk, I don’t mean a measure in the mathematical sense, which requires real numbered values and the additivity property (which I will come to in Sect.  5).

6 Degrees of abnormality, so defined, will meet the conditions for a *negative ranking function* (see, for instance, Spohn, 2012, Sect.  5.2, Smith 2024b, Sect.  4).

7 By substituting similarity spheres for normalcy spheres, a modal theorist can use the same technique to generate degrees of modal risk; the degree of dissimilarity of a proposition P will be equal to the number of similarity spheres throughout which P is false, with the modal risk of P being inversely related to its dissimilarity. On one approach, the smallest similarity sphere will contain just the actual world (Lewis calls this *centering* – see Lewis, 1973, Sect.  1.3). In this case, the actual world will be the only privileged world. On another approach, worlds which differ minimally from the actual world will also be included in the smallest similarity sphere and will also count as privileged (Lewis calls this *weak centering* – see Lewis, 1973, Sect.  1.7). In a normalcy ordering, as discussed above, there is no requirement that the actual world be privileged. It’s important to note that the modal theory will be subject to whatever general problems are associated with the existence of privileged worlds in addition to the specific problems, mentioned above, that stem from the actual world having a privileged status.

8 If W were an infinite set, then this proposition should arguably have a probability of 0. In setting up their unified theory of risk Hirvelä and Paterson assume a finite set of possible worlds and I will, for the most part, follow them in this.

9 If there was just one privileged world, as in the modal theory with a centering constraint (see n7), then we wouldn’t have this result – on the contrary, it would be impossible for both a proposition and its negation to have maximal risk. While this might seem like a good thing, it comes with further baggage – for it will also be impossible for a proposition and its negation to ever have *equal* risk, as the true member of the pair would always have a higher risk than the false member.

10 Degrees of normic risk, so defined, will meet the conditions for what Spohn terms a *two-sided* ranking function. Spohn’s derivation of two-sided ranking functions from negative ranking functions (Spohn, 2012, Sect.  5.2) is an obvious inspiration for the present reinterpretation of normic risk. Degrees of positive normic risk are equivalent to degrees of *normic support* – see Smith (2016, Chap. 7).

11 Could a defender of the modal theory use the same strategy to overcome the objections to their view? A modal theorist could certainly introduce another side to the modal risk scale, following the blueprint laid out here: If a proposition is true at all of the worlds in the smallest similarity sphere then that could be reckoned a higher modal risk than a proposition that is only true at some of these worlds, and if a proposition is true at all of the worlds in the second similarity sphere then this can be reckoned a higher risk again and so on. With this modification, there will no longer be privileged worlds and the actual world will only count as ‘semi-privileged’. This will answer the general problems that arise from the existence of privileged worlds, but not the specific problems that apply to the modal theory, as assessments of risk will still be saddled with presumptions about truth and falsity. On a two-sided interpretation of modal risk, a false proposition could not have a risk any higher than the midpoint, and a true proposition could not have a risk any lower than the midpoint. As a result one could not judge that a proposition has lower than middle risk without committing oneself to its falsity, and could not judge that a proposition has higher than middle risk without committing oneself to its truth. There is more to investigate here, but the modal theory is not my primary concern in this paper.

12 **Table:**

(1)	Pr(P) = Pr((P ∧ Q) ∨ (P ∧ ∼Q))	Logical equivalence
(2)	Pr(P) = Pr(P ∧ Q) + Pr(P ∧ ∼Q)	1, additivity
(3)	Pr(Q) = Pr((P ∧ Q) ∨ (∼P ∧ Q))	Logical equivalence
(4)	Pr(Q) = Pr(P ∧ Q) + Pr(∼P ∧ Q)	3, additivity
(5)	Pr(P ∨ Q) = Pr((P ∧ ∼Q) ∨ (∼P ∧ Q) ∨ (P ∧ Q))	Logical equivalence
(6)	Pr(P ∨ Q) = Pr(P ∧ ∼Q) + Pr(∼P ∧ Q) + Pr(P ∧ Q)	5, additivity
(7)	Pr(P ∨ Q) = Pr(P) + Pr(Q) – Pr(P ∧ Q)	2, 4, 6

13 One might think that this still validates something significantly more general than the original checklist reasoning pattern, as the reasoning will work for normic risk values that are only slightly below 0, and might not be considered ‘low risk’. But the notion of a low risk is, of course, highly context sensitive. Arguably, the only formal requirement on a normic low risk threshold is that it be set below 0 – ensuring that the truth of a proposition has a lower risk than its falsity. Perhaps there are settings in which it is reasonable to set the threshold only slightly below 0, in the same way that there may be settings in which it is reasonable to set a probabilistic low risk threshold only slightly below 0.5. One possible example is in the context of a civil trial, in which elements of a plaintiff’s claim may be dismissed if their probability, given the evidence, is deemed to be lower than 0.5 (see Smith, 2021b). I won’t pursue this further here.

14 An earlier version of this theory is presented in Hirvelä (2025, Sect.  4).

15 Hirvelä and Paterson also introduce a binary accessibility relation on possible worlds (Hirvelä & Paterson, 2024, pp. 12-13) – but assume for ease that all worlds are accessible from the world at which risk scores are calculated. If we let W be the set of all worlds that are a priori possible, we could then use an accessibility relation to encode different ways in which that set might be evidentially restricted. On this approach world x will be accessible from world w just in case x is compatible with the evidence possessed at w. This relation should then figure in the calculation of risk scores; the risk of P, at w, should be calculated by summing the similarity scores of accessible P-worlds and dividing by the sum of the scores of all accessible worlds. The formalism used in earlier sections could be extended in the same way. In this case, each world w will be assigned its own set of normalcy spheres which exhaust the worlds that are accessible from w and contain no worlds which are inaccessible from w (Smith, 2016, Chap. 7). The extended formalism has a range of uses – such as assigning truth conditions to embedded constructions like ‘there is a high risk that P has a high risk’ etc. For present purposes, though, we don’t need to move beyond the simple formalism.

16 This ties in with the issue mentioned in n7 and n9 – namely, whether the actual world should be regarded as the sole occupant of the smallest similarity sphere. Consider all of the worlds that are highly similar to the actual world. Given the set-up in the revolver example, there should be an equal number of these worlds in which the bullet is in each possible location. If these worlds constitute the smallest similarity sphere, then they should all receive the same similarity score. If the same pattern plays out with the larger spheres, then this will be a situation in which every possible location has equal unified risk, and the probabilistic risks and unified risks are the same. An alternative is that there is one smaller similarity sphere, inside this one, which contains the actual world alone. In this case, the actual world would get a higher similarity score and the true location of the bullet would have a higher risk. As explained in the main text, neither approach offers a way of resolving the present problem.

17 The modal and normic theories of risk are sometimes invoked to explain various epistemic judgments (Pritchard, 2015, Sects.  5, 2016; Smith 2021a, Sect.  4). Amongst these are the judgment that lottery beliefs – like the belief that ticket #547 didn’t win the lottery or the belief that ‘7, 32, 14, 8, 11, 4’ won’t be the winning lottery numbers – aren’t justified and cannot constitute knowledge when based purely on the odds involved. While these beliefs have a low probabilistic risk of falsity – lower than that of many beliefs we hold – the modal and normic risk of falsity is high. There will be very similar worlds, and maximally normal worlds, in which #547 wins the lottery and in which ‘7, 32, 14, 8, 11, 4’ are the winning lottery numbers. For the reasons given in the main text, the unified theory cannot, in this case, emulate the predictions of the modal and normic theories and will, on the contrary, give exactly the same predictions as the probabilistic theory.

18 Hirvelä and Paterson may intend to endorse the specific judgment about equiprobable bomb cases while rejecting the more general judgment. But this strikes me as an unstable combination. It’s one thing to reject Pritchard’s judgments wholesale, but if we accept that Bomb 1 really does involve a higher risk than Bomb 2 I think this will have something to do with the perceived *nature* of a lottery and not a mere dissatisfaction with its *size*. Speaking for myself, the kind of thought that would make me most anxious in Bomb 1 is that the lottery must have *some* outcome, and any outcome would be just as normal, and could happen just as easily, as any other. If I were to learn that there were more numbers in the lottery than I’d realised and, thus, more possible outcomes then, while that would be reassuring in one way, it wouldn’t address this basic concern.
